# The orphan nuclear receptor EAR-2 (NR2F6) inhibits hematopoietic cell differentiation and induces myeloid dysplasia in vivo

**DOI:** 10.1186/s40364-018-0149-4

**Published:** 2018-12-07

**Authors:** Christine V. Ichim, Dzana D. Dervovic, Lap Shu Alan Chan, Claire J. Robertson, Alden Chesney, Marciano D. Reis, Richard A. Wells

**Affiliations:** 1 Materials Engineering Division, Lawrence Livermore National Lab, 7000 East Ave, Livermore, CA USA; 2 Nuclear Exploration Inc., Palo Alto, California 94301 USA; 3 Department of Medical Biophysics, University of Toronto, Sunnybrook Research Institute, Toronto, ON M4N 3M5 Canada; 4 Biological Sciences, Sunnybrook Research Institute, Toronto, ON M4N 3M5 Canada; 5 Department of Immunology, University of Toronto, Toronto, ON M5S 1A8 Canada; 6 VCU Medical Centre, Department of Pathology, Richmond, VA 23298 USA; 7 Department of Medicine, University of Toronto, Toronto, ON M5G 2C4 Canada; 8 Department of Medical Oncology, Myelodysplastic Syndromes Program, Toronto Sunnybrook Regional Cancer Centre, Toronto, ON M4N 3M5 Canada; 9 Department of Laboratory Hematology, University Health Network, Toronto, ON M5G 2C4 Canada

**Keywords:** Myelodysplastic syndrome, Stem cell, Mouse model, Hematopoiesis, Bone marrow transplant, Clonogenicity, Differentiation, Progenitor cell, Nuclear receptor

## Abstract

**Background:**

In patients with myelodysplastic syndrome (MDS), bone marrow cells have an increased predisposition to apoptosis, yet MDS cells outcompete normal bone marrow (BM)-- suggesting that factors regulating growth potential may be important in MDS. We previously identified v-Erb A related-2 (EAR-2, NR2F6) as a gene involved in control of growth ability.

**Methods:**

Bone marrow obtained from C57BL/6 mice was transfected with a retrovirus containing EAR-2-IRES-GFP. Ex vivo transduced cells were flow sorted. In some experiments cells were cultured in vitro, in other experiments cells were injected into lethally irradiated recipients, along with non-transduced bone marrow cells. Short-hairpin RNA silencing EAR-2 was also introduced into bone marrow cells cultured ex vivo.

**Results:**

Here, we show that EAR-2 inhibits maturation of normal BM in vitro and in vivo and that EAR-2 transplant chimeras demonstrate key features of MDS. Competitive repopulation of lethally irradiated murine hosts with EAR-2-transduced BM cells resulted in increased engraftment and increased colony formation in serial replating experiments. Recipients of EAR-2-transduced grafts had hypercellular BM, erythroid dysplasia, abnormal localization of immature precursors and increased blasts; secondary transplantation resulted in acute leukemia. Animals were cytopenic, having reduced numbers of erythrocytes, monocytes and granulocytes. Suspension culture confirmed that EAR-2 inhibits granulocytic and monocytic differentiation, while knockdown induced granulocytic differentiation. We observed a reduction in the number of BFU-E and CFU-GM colonies and the size of erythroid and myeloid colonies. Serial replating of transduced hematopoietic colonies revealed extended replating potential in EAR-2-overexpressing BM, while knockdown reduced re-plating ability. EAR-2 functions by recruitment of histone deacetylases, and inhibition of differentiation in 32D cells is dependent on the DNA binding domain.

**Conclusions:**

This data suggest that NR2F6 inhibits maturation of normal BM in vitro and in vivo and that the NR2F6 transplant chimera system demonstrates key features of MDS, and could provide a mouse model for MDS.

**Electronic supplementary material:**

The online version of this article (10.1186/s40364-018-0149-4) contains supplementary material, which is available to authorized users.

## Background

Myelodysplastic syndrome (MDS) is a bone marrow failure syndrome characterized by peripheral blood cytopenias, apoptosis of bone marrow hematopoietic progenitors, abnormal blood cell morphology (dysplasia) and a marked propensity to progress to acute leukemia [1–4]. MDS bone marrow (BM) is characterized by reduced numbers of mature progeny and maturing progenitors that exhibit impaired clonogenicity [5] along with a high rate of apoptosis [6–8]. Paradoxically, MDS comes to dominate the bone marrow at the expense of residual normal hematopoiesis and thereby causes disease. The difficulty of culturing MDS cells *ex vivo* [9] and in xenotransplant models [10–17], and the lack of mouse models of MDS that recapitulate all aspects of disease [18–20] hampers study of this condition. Generation of clinically accurate mouse models of MDS remains an open challenge in hematological oncology research. While multiple genetically engineered mouse models of MDS have been developed [21–31], the diverse genetics and phenotypic heterogeneity of the human disease are not accurately represented by these models, and many of the models do not recapitulate all aspects of clinical presentation.

The quandary of MDS’ competitive advantage, but poorer growth, could be resolved if the myelodysplastic syndrome population of cells contains two or more distinct populations -- genetically identical but developmentally and/or epigenetically distinct. For example, if myelodysplastic syndrome marrow were composed of populations of rare stem and plentiful non-stem MDS progenitor cells, a gene that could function to inhibit differentiation of different cell populations could phenocopy MDS. An inhibition of differentiation of the putative MDS-stem cells would lead to expansion of the stem cell compartment, giving those stem cells a clonogenic advantage over normal stem cells. If this was coupled with an impairment of differentiation in the progenitor cell population, this gene would simultaneously block the ability of non-stem MDS progenitor cells to form colonies, differentiate and mature into functional blood cells while also giving those colonies the ability to clonally dominate the bone marrow.

This cancer stem cell based hypothesis would explain the decreased growth ability of the bulk, primarily non-stem population in MDS while simultaneously explaining the competitive advantage of MDS stem cells over normal marrow stem cells. To test this hypothesis, we determined if a gene that we suspected decreased differentiation and increases stem cell self-renewal based on a screen we previously performed [32] could be involved in the pathogenesis of MDS.

In our previous work comparing the transcriptional profiles of individual proliferative and non-proliferative leukemia cells, we found that mRNA transcripts of v-Erb A related-2 (EAR-2, NR2F6) are more abundant in proliferative AML cells compared to AML cells that spontaneously growth arrest [32], suggesting that EAR-2 may increase proliferative ability, self-renewal and/or block differentiation. Indeed, in normal mouse hematopoietic tissue, we found the highest expression of *EAR-2* in long-term repopulating HSCs, declining with differentiation [32]. *EAR-2* expression is increased in the marrow of patients with AML and myelodysplastic syndrome (MDS) compared to normal BM [32], and silencing of *EAR-2* expression in AML cell lines results in terminal differentiation and apoptosis [32], showing that EAR-2 regulates differentiation also in malignancies. While we were the first group to identify a role for EAR2 in AML [32], other groups have since shown that EAR-2 is upregulated in breast cancer [33], colorectal cancer [34], cervical cancer [35], ovarian cancer [36] and bladder cancer [37].

The role of EAR-2 in hematopoiesis has been previously unstudied. Indirect evidence suggests that EAR-2 acts in hematopoiesis by repressing the key hematopoietic transcription factor Runx1 [38]. The high expression of EAR-2 in patients with MDS (*n* = 12), AML (*n* = 15) and CMML (*n* = 10) [32], along with the ability of *EAR-2* to regulate differentiation in leukemia cell lines and its pattern of expression in the normal hematopoietic hierarchy suggests a link between EAR-2, stem/progenitor cell dysfunction and the pathogenesis of MDS. Therefore, we wished to investigate here whether EAR-2 expression is merely correlative with undifferentiated cell states, or whether expression had functional consequences to the undifferentiated state of normal and/or malignant tissue.

We postulated that over-expression of EAR-2 in vivo would inhibit hematopoietic differentiation as it does in leukemia cell lines, and thus result in myeloid dysplasia, as characterized by the cardinal features of MDS: stem cell competitive advantage, dysplastic hematopoiesis, peripheral blood cytopenias, and progression to acute leukemia. We used a retroviral construct to over-express EAR-2 exogenously in normal murine bone marrow cells in a chimeric setting to test competitive advantage. We then cultured marrow from these chimeric mice to test the ability of the cells to differentiate ex vivo, while monitoring the animals (either in primary or secondary transplants) to see if they would progress to frank leukemia.

In vivo*,* over-expression of EAR-2 in bone marrow cells resulted in development of a pre-leukemic state resembling MDS that culminated in AML. The effects of EAR-2 on hematopoiesis appear to be mediated by impairment of differentiation and extended proliferative capacity caused by regulation of gene expression in a DNA-binding-dependent fashion, and in part by modulating histone acetylation. Our study is the first to establish that the orphan nuclear receptor EAR-2 is able to induce a pre-leukemic condition that is caused by impaired regulation of hematopoietic cell differentiation and lineage commitment. This suggests that the paradoxical observation that the MDS bone marrow clone can be both inefficient in survival and hematopoiesis but can proliferate sufficiently to out-compete normal bone marrow may be explained, at least in part, by genetic mutations that cause an impairment in differentiation, that has differing consequences in two distinct cellular compartments.

## Methods

### Animals

C57BL/6 mice were obtained from the Jackson Laboratory (Bar Harbor, ME) and stored in a specific pathogen-free facility at Sunnybrook Research Institute. All work was done in accordance with the Sunnybrook Research Institute Animal Care and Use Guidelines.

### Generation of retrovirus

EAR-2 retrovirus was generated as described [32, 39].

### Bone marrow transduction

Bone marrow was transduced as described [40].

### Hematopoietic stem cell transplant chimeras

Primary chimerical transplants were performed as follows: 5FU-primed C57Bl/6 bone marrow cells were transduced with either GFP or EAR-2-IRES-GFP previously cloned in to the MMP vector [32] as described above. Transduced cells were then isolated by FACS based on GPF expression. Animals were then injected with a mixture of 7.5 × 10^4^ cells mock-transduced donor cells and either 2.5 × 10^4^ cells EAR-2-GFP transduced (experimental) or GFP transduced cells (control). Recipients of chimerical bone marrow were harvested either at 4–6 weeks (early time points) or at 12–16 weeks (late time points). For the competitive transplant experiment the percentage of transduced cells was determined based on expression of GFP using flow cytometry.

Secondary transplantation experiments were performed as follows: recipient animals that received chimerical grafts were harvested at 12 weeks post-transplant, and bone marrow was transplanted into another lethally irradiated mouse by tail-vein injection.

### Histological sections and cytospins

Histological sections were performed as previously described [40]. Bone tissues were decalcified following fixation before further processing. Cytospins preparations were stained with May-Gruwald and Giemsa stains. Percentage blasts were determined by counting a minimum of 100 bone marrow cells, in three distinct fields of view, per animal evaluated.

### Peripheral blood counts

Blood from bone marrow transplant recipients was obtained at 4 weeks post-transplant. Alternatively, moribund animals were bled by cardiac puncture just prior to death. To give matched data, a GFP control animal was analysed with every EAR-2 moribund animal analysed. Blood was collected in a heparinized capillary tube and hematological parameters were acquired on a Hemavet analyser.

### Flow cytometry

For analysis by flow cytometry red blood cell depleted bone marrow cells were stained with one of more of the following: biotin CD3, biotin CD45R/B220 (RA3-6B2), biotin CD11b (M1/70), biotin erythroid marker (TER-119), biotin Ly-6G (RB6-8C5), c-kit APC, sca-1 PE-Cy7 and either CD34 PE or CD49b PE (all eBioscience) in the dark. Bone marrow was washed once and incubated with streptavidin PE-Cy5 for 20 min in the dark. Bone marrow was washed twice and analysed using flow cytometry on a Becton Dickinson LSR II. All samples analysed were gated based on FSC/SSC and GFP+ cells.

### Methylcellulose colonies

Following bone marrow transduction, GFP positive cells were isolated by FACS and plated in methylcellulose medium (Methocult GF 3434, Stem Cell Technologies). Colony formation was evaluated after 10–14 days; clusters containing more than 30 cells were scored as a colony. Secondary colony formation was tested by harvesting entire primary colony cultures, washing the cells two times with PBS, and plating 10,000 cells in methylcellulose a second time. Secondary colonies were enumerated 12–14 days following secondary plating. Cells/colony were calculated by dividing the total number of cells obtained per dish by the total number of colonies in that dish. We confirmed using fluorescent microscopy that all colonies continued to maintain transgene expression.

### Ex vivo suspension culture

Following transduction of mouse bone marrow with MMP-GFP or MMP-EAR2-IRES-GFP, cells were placed unsorted into IMDM with 5% FBS, 10% *v*/v IL-3 conditioned medium from WEHI-3 cells, 1 ng/mL IL-6 and 3% v/v c-kit ligand conditioned medium. Following ten days of culture the cells were washed twice with PBS, stained with either fluorescently labelled c-kit or with fluorescently labelled CD11b and GR-1, and analysed by flow cytometry.

### Generation of shRNA against EAR-2

shRNA was generated and knockdown was confirmed by Western Blotting as previously described [32, 39]. This construct routinely gave knockdown of ~ 75% in our hands in hematopoietic cell lines. Due to limited sample availability, knockdown was confirmed at the mRNA level on sorted bone marrow cells by qPCR (data not shown).

## Results

### EAR-2 transplant chimeras exhibit perturbed hematopoietic differentiation and dysplastic hematopoiesis

To model the effects of EAR-2 over-expression that we previously observed in AML and MDS patient samples [32], we performed adoptive transfer of EAR-2-transduced hematopoietic cells into lethally irradiated syngeneic recipients in a competitive repopulation model. EAR-2 was over-expressed by transducing bone marrow cells with a retrovirus that expresses EAR-2 and EGFP as a bicistronic transcript [32]. Grafts contained a mixture of cells transduced with EAR-2 or control virus (~ 25%) and untransduced cells (~ 75%). Early engraftment was evaluated in 16 recipients at 4 weeks post-bone marrow transplant.

**Fig. 1:**
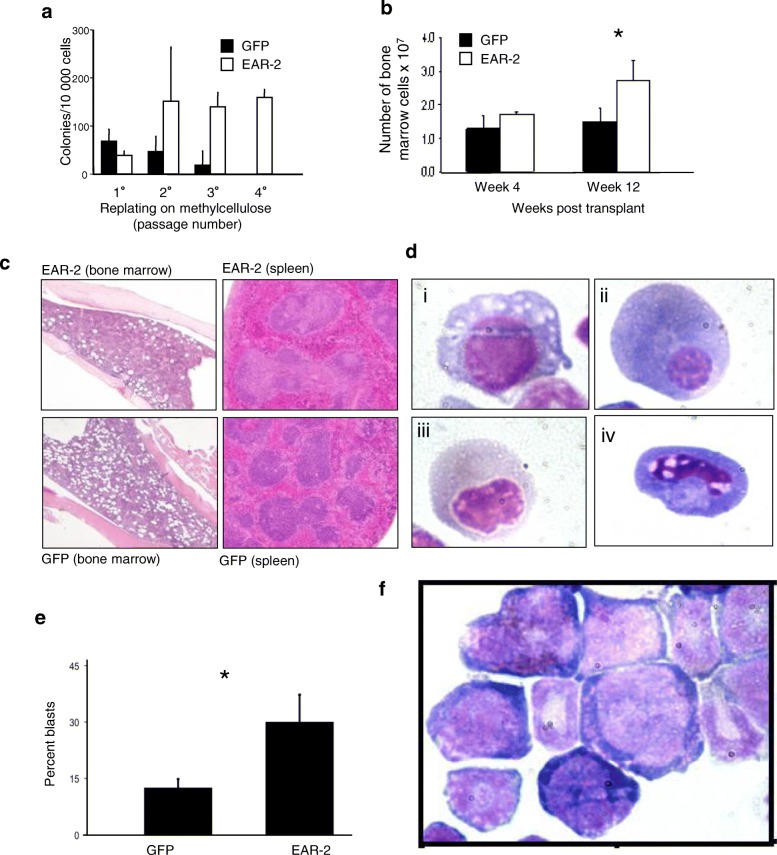
EAR-2 over-expression chimeras develop myeloid dysplasia. **a** Serial replating ability was analyzed in methylcellulose medium. **b** Bone marrow cellularity post-transplant. **c** Photomicrographs of the bone marrow and spleen post-transplant. **d** Dysplasia of the erythroid lineage observed in EAR-2 BM transplants. **e** Enumeration of the number of blasts and promyelocytes in bone marrow of transplanted animals. **f** Photomicrograph of cystospin of blast cells

Engraftment was successful in 6 of 8 recipients of EAR-2 transduced bone marrow cells and 8 of 8 recipients of GFP transduced cells. In all of these recipients, there was an increase in the proportion of GFP^+^ bone marrow cells in comparison to the input proportion (Additional file 1: Figure S1a) whereas an analogous increase was not seen in GFP-only controls, indicating that EAR-2-transduced cells had a competitive advantage over untransduced cells in repopulation. Correspondingly, EAR-2-transduced cells recovered from recipients had a striking increase in replating-ability relative to GFP-only-transduced cells (Fig. 1a) and there was an increase in overall bone marrow cellularity in the EAR-2 chimeras, a difference that was more marked in a second cohort of primary recipients analyzed at 12 weeks post-BMT (Fig. 1b).

Histological examination showed bone marrow hyper-cellularity and distortion of splenic architecture, with expansion of myeloid cells in the germinal centre regions (Fig. 1c). The cytomorphological appearance of bone marrow cells was abnormal, with numerous dysplastic erythroid precursors (Fig. 1d). Additionally, we observed pancytopenia in EAR-2 BMT recipients 12 weeks post-transplant (Table 1). In contrast to GFP controls, where clusters of primitive cells were found only in paratrabecular locations, EAR-2 BMT recipients exhibited such clusters also in the intertrabecular region (Additional file 1: Figure S1b, c) – this phenomenon, known as abnormal localization of immature precursors (ALIP), is seen in myelodysplastic syndrome, where it is a predictor of aggressive disease behaviour [41]. EAR-2 recipients also had a higher percentage of morphologically primitive cells (myeloblasts and promyelocytes) compared to controls (Fig. 1e) with the morphology shown in Fig. 1f. The observations of pancytopenia, hypercellular bone marrow, increase in primitive cells, and ALIP in the bone marrow comprise a phenotype similar to MDS. This phenotype was observed in all animals that successfully engrafted with EAR-2 transduced bone marrow chimeras. However, 2/8 animals in the EAR-2 cohort did not engraft, meaning no expression of GFP+ cells (EAR-2 expressing cells) were observed either in the bone marrow, spleen or peripheral blood.

**Table 1:** Blood counts of EAR-2 transplant chimeras

	4 week post-BMT		12 week post-BMT	
	GFP (*n* = 7)	EAR-2 (*n* = 8)	GFP (*n* = 4)	EAR-2 (*n* = 4)
Hgb (g/L)	135.0 (125–144.5)	134.6 (132.8–141.7)	139.0 (134–144)	58.0 (57–59)
PLT (10^^9^/L)	507.4 (334–567)	517.5 (478–562.3)	784.0 (559–1009)	317.0 (236.5–397.5)
WBC (10^^9^/L)	3.9 (1.9–5.3)	3.1 (2.3–3.6)	17.4 (15.2–19.7)	7.8 (6.3–9.2)

### Secondary transplantation of EAR-2 bone marrow chimeras results in acute leukemia

**Fig. 2:**
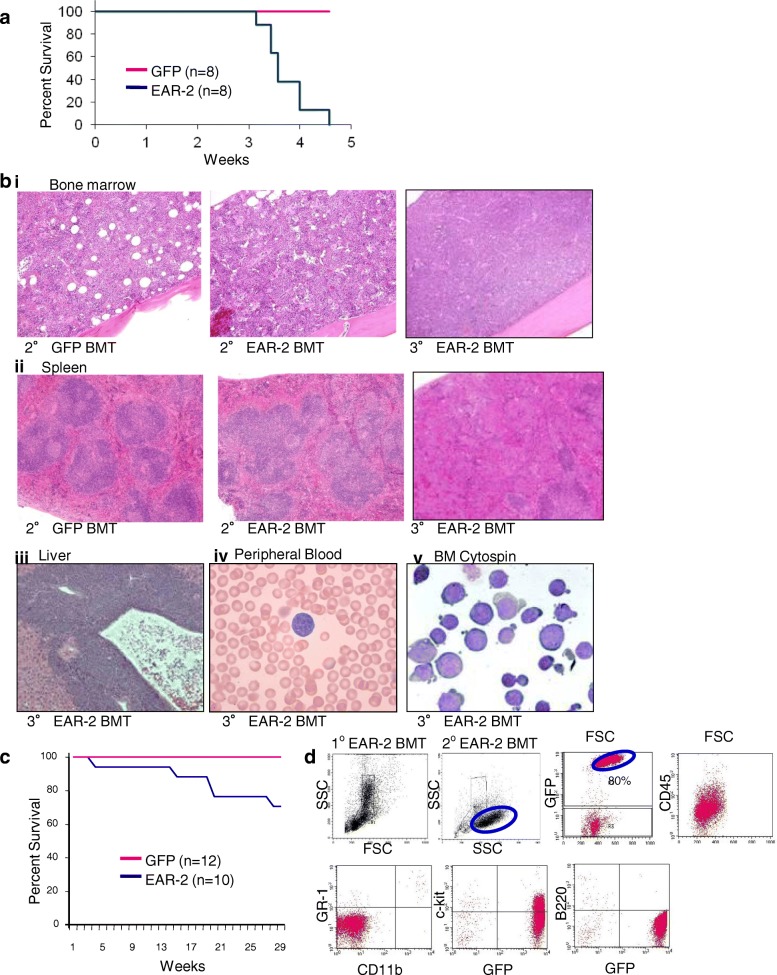
Secondary transplantation of EAR-2 bone marrow chimeras results in acute leukemia. **a** Survival of secondary transplant recipients (*n* = 8 GFP and *n* = 8 EAR-2). Animals that received secondary-transplantation with EAR-2+ BM showed infiltration by leukemia cells in the **(b i)** bone marrow, **(ii)** spleen, **(iii)** liver and **(iv)** peripheral blood. These effects were even more pronounced in tertiary transplant recipients. **(b v)** Morphology of the leukemia blast cells is shown. **c** Survival curve for bone marrow transplant chimera not receiving secondary transplantation. **d** Immunophenotype of the blast cells is shown

To investigate the effect of EAR-2 over-expression on the ability of stem cells to differentiate and repopulate the bone marrow, we conducted secondary bone marrow transplants. Twelve weeks after initial transplantation, 1 × 10^6^ whole bone marrow was transferred from primary recipients into a second, lethally irradiated host. All secondary recipients developed acute leukemia and became moribund between 3 and 4 weeks of transplant (*n* = 15 total recipients from 13 donor chimeras, i.e., most donor bone marrow was transplanted in to a single recipient, but some donor animals were transplanted in two recipient animals; animals that did not engraft were not used in secondary transplant experiments, these data consisted of two independent experimental cohorts). Figure 2a shows the survival data from one of these cohorts (*n* = 8 EAR-2 recipients and *n* = 8 GFP recipients).

The leukemia was characterized by hematopoietic failure, massive splenomegaly, and infiltration of bone marrow (Fig. 2b iii), spleen (Fig. 2b ii), liver (Fig. 2b iii), and peripheral blood (Fig. 2b iv). Blasts had an erythroid morphology as shown in Fig. 2b v. The immunophenotype of the leukemia in bone marrow was GFP+, CD45^low^, c-kit^low^, GR-1-, CD-11b-, and B220- (Fig. 2d). Furthermore, the leukemia was transplantable serially into non-irradiated tertiary and quaternary recipients, which also died rapidly of the leukemia (Fig. 2b). We then determined whether secondary transplantation was necessary for the development of leukemia, or whether primary recipient animals would develop leukemia after longer latency. We observed that after a variable latency some animals developed acute leukemia (Fig. 2c).

These data demonstrate that, like MDS, the disease that develops in mice that over-express EAR-2 tends to evolve into acute leukemia. Furthermore, these data confirm that the leukemia induced by EAR-2 over-expression is transplantable (Fig. 2b), and therefore contains leukemia initiating cells.

### Over-expression of EAR-2 in vivo inhibits hematopoietic differentiation

**Fig. 3:**
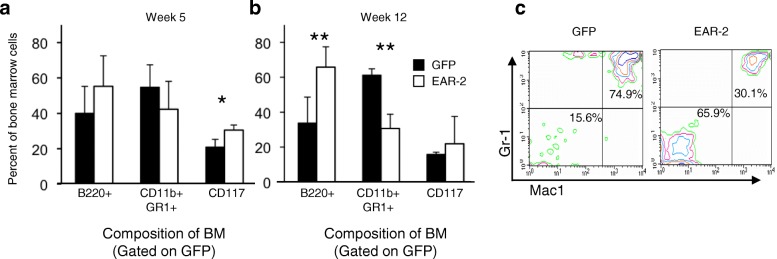
Over-expression of EAR-2 in vivo inhibits progenitor cell differentiation. Flow cytometry on bone marrow from transplant chimeras using lineage markers of B-cells, granulocytes and progenitor cells, at **a** 5 weeks, **b** 12 weeks, and **c** a contour map of datum from a representative animal at 12 weeks

To assess the role of EAR-2 on hematopoietic lineage specification and differentiation in vivo, we analyzed the composition of primary chimeric bone marrow recipients using flow cytometry. We observed a significant decrease in the proportion of granulocytes (CD11b^+^, GR-1^+^ cells) (Fig. 3a, b and c). These data are consistent with a model in which unregulated expression of EAR-2 perturbs hematopoietic differentiation of the myeloid lineages. Unexpectedly, the EAR-2 overexpressing fraction of the bone marrow contained a significant increase in the proportion of B220^+^ cells. We did not confirm that B220+ cells were B-cell progenitors. Given that EAR-2 is a transcriptional regulator, we do not know if the increase B220 expression reflects bona fide changes to the lineage composition of the bone marrow or whether B220 is a direct target of EAR-2 and the antigen is expressed promiscuously.

### Expression of EAR-2 negatively regulates erythroid and myeloid differentiation of bone marrow cells

We hypothesized that down-regulation of EAR-2 is a key step in the process of hematopoietic differentiation, and that unregulated expression of EAR-2 would block differentiation. We tested this hypothesis by manipulating EAR-2 expression in normal murine bone marrow cells in vitro so that we could investigate the role of EAR-2 in a cell intrinsic manner. We first sought to characterize the effect of forced expression of EAR-2. In contrast to earlier experiments that examined cell differentiation in vivo after cell chimeras were transplantated into syngeneic hosts, these experiments looked at the ability of the cells to differentiate in a controlled in vitro environment that is not confounded by factors such as the immune system, engraftment ability and other confounds of an in vivo environment.

**Fig. 4:**
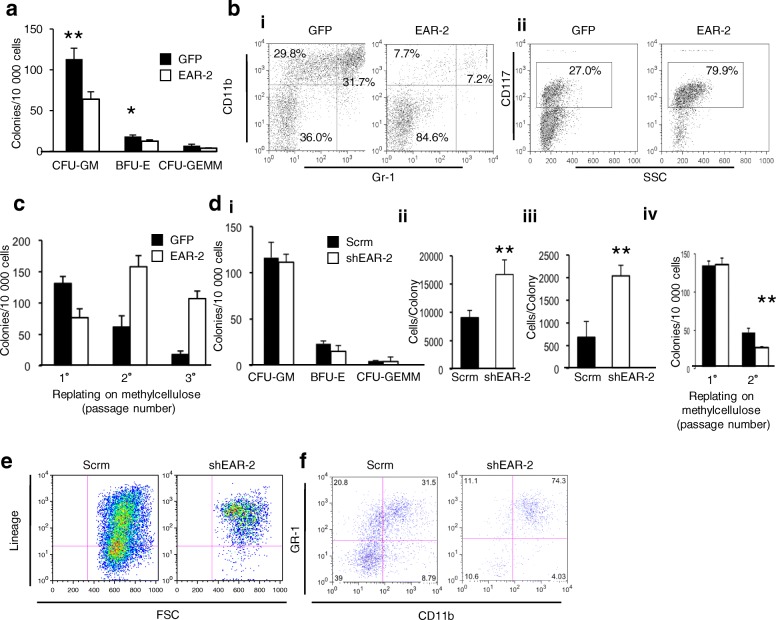
Over-expression of EAR-2 in vitro inhibits hematopoietic differentiation while knockdown increases hematopoietic differentiation. Bone marrow transduced with either EAR-2 or empty vector (GFP) was analyzed for **a** colony formation in methylcellulose medium, (**b i and ii**) and differentiation ability in suspension culture followed by immunophenotypic analysis using flow cytometry. **c** Serial colony formation assays of bone marrow ex vivo transduced with EAR-2 shows an increased proliferative capacity ex vivo. Silencing of murine BM with shRNA did not effect (**d i**) colony number, but greatly increased colony size in conditions favoring (**d ii**) multilineage and (**d iii**) erythroid differentiation. (**d iv**) Silencing of EAR-2 decreased serial colony forming ability of bone marrow. **e** Gene silencing of EAR-2 depleted bone marrow of undifferentiated cells (the lineage negative fraction) in short-term culture, **f** such that the cells differentiated into granulocytes

Colonies derived from EAR-2 transduced bone marrow cells were significantly smaller than controls, showing a 49.4% decrease in the average number of cells per colony (Additional file 2: Figure S4a). EAR-2-transduced bone marrow cells in vitro yielded 42.9% fewer myeloid and 34.6% fewer erythroid colonies in comparison to GFP controls (Fig. 4a). Furthermore, EAR-2-overexpressing bone marrow cells maintained in suspension culture containing myeloid promoting growth factors retained an immature immunophenotype, and maintained cell surface expression of CD117 while failing to activate expression of CD11b and Gr-1 (Fig. 4b i and 4b ii). These data demonstrate that EAR-2 expression negatively regulates the differentiation of erythroid and myeloid cells. To show that this is not an effect dependent on strain background, we repeated this experiment in bone marrow from Balb/c mice, and likewise observed a decrease in the number of colonies derived from bone marrow transfected with EAR-2 in Balb/c mice as we did in C57BL/6 animals (Additional file 2: Figure S4b).

### Expression of EAR-2 regulates proliferative capacity

Differentiation encompasses both phenotypic maturation as well as loss of proliferative ability. Hence, we assessed the effect of EAR-2 expression on the replicative capacity of hematopoietic cells by means of a colony-forming replating assay. In contrast to experiments in Fig. 1a, that looked at replating of bone marrow in post-transplant animals, we wished to assess replating on freshly transduced bone marrow cells to observe the effect of EAR-2 without confounding factors or selective pressure that could contribute to additional mutations, epigenetic changes or selection. While GFP transduced hematopoietic cells exhausted their clonogenic potential after two rounds of re-plating, serial re-plating of EAR-2 overexpressing- bone marrow revealed extended proliferative ability (Fig. 4c). These observations suggest that constitutive expression of EAR-2 not only inhibits maturation and differentiation, but also enhances proliferative potential; thus we then tested whether reduced levels of EAR-2 would increase differentiation and reduce proliferative potential.

### Knockdown of EAR-2 induces differentiation of bone marrow cells

Our data indicate that forced overexpression of EAR-2 has major effects on hematopoiesis. Since EAR-2 expression can be detected in very early hematopoietic cells [32] we conjectured that this protein might also play a physiological role in regulating hematopoietic differentiation.

To investigate this possibility, we assessed the effects of silencing EAR-2 expression with shRNA on colony formation in vitro. We used an shRNA construct that routinely gave knockdown of ~ 75% in our hands in hematopoietic cell lines. Due to limited sample availability, knockdown was confirmed at the mRNA level on sorted bone marrow cells by qPCR (data not shown). While silencing of EAR-2 did not significantly reduce the number of colony forming units (Fig. 4d i), it did significantly increase the colony size (Fig. 4d ii and iii, and Additional file 2: Figure S4c). In the secondary re-plating assay, we observed a significant decrease in secondary colonies in cells in which EAR-2 expression was silenced (Fig. 4d iv), which is consistent with lower proliferative capacity, inhibition of self-renewal and eventual loss of the stem cell fraction.

We next assessed the effects of EAR-2 on undifferentiated hematopoietic cells by analysing the effects of silencing of EAR-2 on maintenance of stem and progenitor cells in 5-FU treated bone marrow cells in culture. Silencing of EAR-2 resulted in a reduction in stem cells as shown by depletion of undifferentiated lineage negative cells (Fig. 4e and Additional file 2: Figure S4d), and concurrent increase in cells expressing the myeloid markers CD11b and Gr-1 surface antigens, suggesting that these cells had differentiated into cells of the neutrophil lineage (Fig. 4f). These observations were confirmed by examination of cytomorphology of the bone marrow cultures: EAR-2 silenced populations were comprised predominantly of mature neutrophils (Additional file 2: Figure S4e). Taken together, these results establish that exogenous expression of EAR-2 blocks myeloid maturation, and support the idea that EAR-2 is a negative regulator of hematopoietic differentiation.

### EAR-2 functions as a transcriptional repressor in a DNA binding dependent manner

**Fig. 5:**
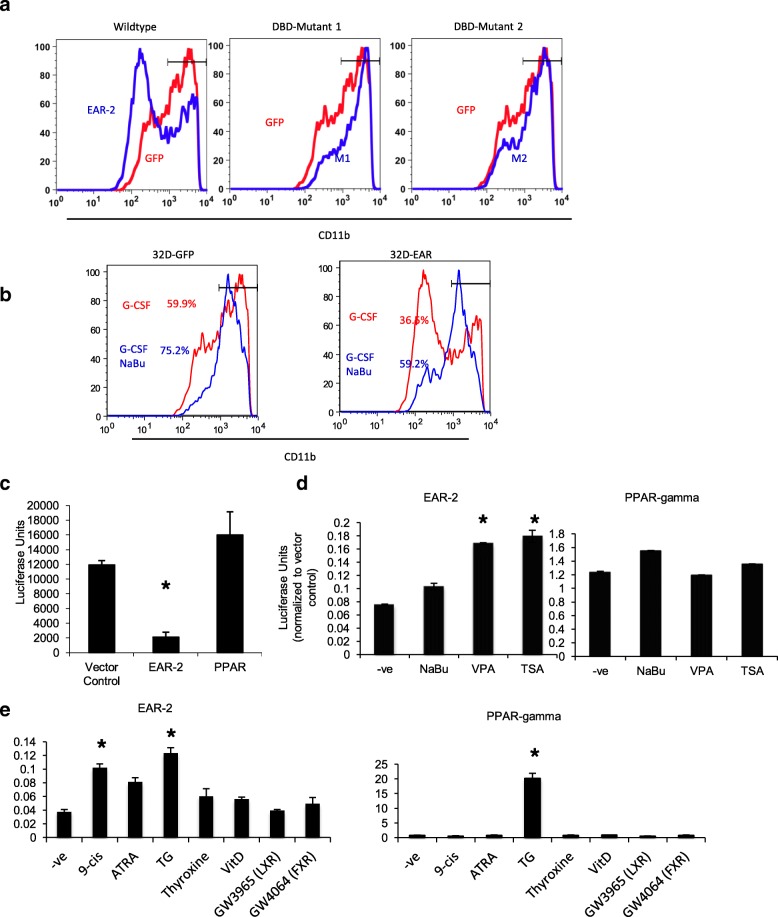
Inhibition of differentiation is DNA-binding and HDAC dependent. **a** Transduction of 32D cells with an EAR-2 mutant that could not bind DNA or with the HDAC inhibitor **b** sodium butyrate rescued ability of EAR-2 to inhibit differentiation. **c** Transfection of EAR-2 showed repression of luciferase activity, **d** that could be de-repressed with HDAC inhibitors. **e** Ability of known nuclear receptor ligands to activate luciferase activity was tested using a hybrid construct containing the EAR-2 ligand binding domain. This was contrasted with a hybrid construct containing the PPARɣ ligand binding domain. (* indicates *p* < 0.05 compared to negative control)

Nuclear receptors may possess DNA binding-dependent as well as DNA binding-independent function. To examine whether the ability of EAR-2 to inhibit differentiation is DNA-binding dependent we used constructs of EAR-2 with mutations that disrupt the DNA binding domain [42], specifically in the P-box (M1) and D-box (M2) of the zinc finger domains. We observed that mutation of either the P-box or the D-box abrogated the ability of EAR-2 to inhibit differentiation of 32Dcl3 cells (Fig. 5a) suggesting that this phenotype is dependent on the DNA-binding ability of EAR-2, and establishing that this effect is not simply a squelching phenomenon resulting from sequestration of nuclear receptor cofactors by EAR-2.

It has been reported that EAR-2 exerts its regulatory effects primarily as a transcriptional repressor [43]. The repressor activity of many nuclear receptors is mediated by recruitment of corepressors with histone deacetylase (HDAC) activity; we therefore evaluated the importance of this mechanism in the effects of EAR-2 on hematopoiesis.

32D cells that over-expressed EAR-2 (32D-EAR-2) were incubated with the non-specific HDAC inhibitor sodium butyrate prior to treatment with G-CSF which potently induces differentiation to granulocytes. Whereas, non-treated 32D-EAR-2 cells failed to differentiate in response to G-CSF, sodium butyrate pretreated 32D-EAR-2 cells showed recovery of G-CSF induced differentiation as indicated by cell surface CD11b expression (Fig. 5b). Thus, HDAC-mediated transcriptional repression may account for at least part of the mechanism by which EAR-2 impairs hematopoietic differentiation.

In order to test the mechanism of EAR-2 transcriptional regulation further, we constructed a hybrid receptor, comprising the EAR-2 ligand-binding domain fused to the Gal4 DNA-binding domain. The hybrid receptor was co-transfected into HeLa cells along with a reporter plasmid in which a Gal4 regulatory sequence is placed upstream of a luciferase gene running off a constitutive promoter. We observed reduced luciferase activity in transfected cells, suggesting that EAR-2 acts as a strong transcriptional repressor (Fig. 5c). We also observed that treatment with multiple HDAC inhibitors, such as valproic acid, and trichostatin A, de-repressed the transcriptional repression induced by EAR-2 (Fig. 5d). Identification of a ligand for EAR-2 that could modify its activity could be of potential therapeutic interest. We used the hybrid EAR-2 receptor to survey a number of common nuclear receptor ligands for their ability to activate transcription (Fig. 5e). We did this to test the feasibility of this construct to screen small molecule agonists for cross reactivity with the EAR-2 ligand binding domain. We showed that troglitazone (TG) and 9-cis retinoic acid (9-cis) were very weakly able to de-repress the transcriptional repression induced by EAR-2, albeit the effect was very small in comparison to the activation that troglitazone induced of its receptor PPARɣ. These data suggest that it might be possible to modify the activity of EAR-2 using small molecules that de-repress EAR’s transcriptional activity, and that it is feasible to screen small molecule that bind to and modify EAR-2 activity using a hybrid EAR-2 construct.

## Discussion

We hypothesized that the central conundrum of MDS-- that cells derived from the MDS clone are difficult to culture and ineffective in sustaining hematopoiesis in the periphery, but nonetheless attain clonal dominance of the bone marrow-- could be explained by impaired differentiation in among stem and progenitor cells. To study this, we tested whether a gene that impairs differentiation of stem and progenitor cells could recapitulate the clinical phenotype of MDS. We have previously shown that expression of EAR-2 correlates with the growth capacity of leukemia cells, inhibits differentiation of cell lines in vitro and is over-expressed in AML and MDS patient bone marrow samples [32]. Indeed, we found that unregulated expression of EAR-2 in bone marrow cells resulted in a condition that resembled myelodysplastic syndrome (Fig. 1) and culminated in acute leukemia (Fig. 2). This suggests a putative resolution to this paradox: while partially differentiated MDS progenitor cells are unable to expand and differentiate to mature lineages, the MDS stem cell could out-compete normal hematopoietic progenitors because of a selective advantage at the stem cell level.

Progress in understanding MDS has been hampered by the lack of suitable cell lines or animal models for this disease. A mouse model that accurately recapitulates the essential qualities of MDS – stem cell competitive advantage, dysplastic hematopoiesis, peripheral blood cytopenias, and progression to acute leukemia – would be tremendously valuable for investigations of the pathological mechanisms of these qualities and for preclinical testing of new MDS therapies. The EAR-2 transplant chimera has several qualities that recommend it as a model of MDS. The first of these is prima facie plausibility – since EAR-2 is commonly overexpressed in MDS and AML, it is not unexpected that its overexpression might lead to MDS in the mouse. Second, the essential elements of MDS – dysplasia, bone marrow hypercellularity, and progression to acute leukemia – are faithfully recapitulated in the model. Third, in contrast to germline transgenic mouse models, our model has mixed hematopoietic chimerism and therefore reflects the competitive aspect of myelodysplastic hematopoiesis, in which normal and MDS HSCs co-exist. Finally, the long latency preceding the development of leukemia in our model provides opportunity for the study both of the biology of progression of MDS, and of therapeutic interventions.

The majority of chimeric animals that undergo secondary transplantation developed leukemia (Fig. 2). These animals meet the criteria for classification of nonlymphoid leukemia including > 20% accumulation of immature forms/blasts in the bone marrow, rapid mortality, effacement of normal hematopoiesis as characterized by anemia, thromobocytopenia, and leukopenia, increase of hematopoietic cells in the spleen as demonstrated by the effacement of the splenic architecture, infiltration of immature forms/blasts in the blood and liver.

We conjecture that the reason secondary transplantation induces rapid development of leukemia is that reconstitution during transplantation induces a temporary increase in the rate of stem cell divisions in order to achieve HSC expansion. The increased rate of HSC divisions allows for perturbations in HSC dynamics to be resolved in a shorter period of time than had primary recipients been observed for extended periods of time. To test this hypothesis, we observed EAR-2 transplant chimeras over a period of 29 weeks, observing the spontaneous development of leukemia in a portion of the recipients Fig. 2c. These animals were transplanted with a graft consisting of 10% transduced cells, the low percentage of transduced cells may explain the long latency required for the development of leukemia. Nevertheless, the long duration of the latency phase preceding the development of AML in EAR-2 transplant chimeras suggests that a second mutational event may be required to initiate AML in these animals.

It might, at first, appear like a contradiction that EAR-2 over-expression causes a complete block of leukemia cell differentiation (Fig. 2d shown by Gr-1-CD11b-and B220- in other words, almost no GR-1 + CD-11b + and B220+ cells were detected), whereas Fig. 3a shows that EAR-2 only partially inhibits myeloid cell differentiation (about 40% GR-1 + CD-11b + and 60% B220+ cells were detected in bone marrow cells ex vivo that overexpressed EAR-2). While, the data from these two figures might seem contradictory, these data are not because the figures describe two different pathophysiological states. Figure 2d describes a leukemic state, a state in which one cell population has had time to evolve and achieved clonal dominance (the immunophenotype of this population is Gr-1-CD11b-and B220-). On the other hand, Fig. 3a. describes the role of EAR-2 in a pre-leukemia state, where multiple cell populations are present and potentially effected by over-expression of EAR-2. Our data does not describe the relationship between these two populations: we do not know if the leukemia cells are monoclonal, and hence arising from a subsequent mutation in a progenitor cell that is already predisposed to have inhibited terminal differentiation; or whether these cells are polyclonal arising in a separate population that is negative for GR-1, CD-11b or B220 that has a competitive advantage. What our data does demonstrate is that while some degree of hematopoietic differentiation may be permitted with over-expression of EAR-2, ultimately, in the leukemic state one (or more clones) that is Gr-1-CD11b-and B220- comes to dominate the bone marrow causing leukemia.

In ex vivo bone marrow culture (Fig. 4), over-expression of EAR-2 led to reduction in the number of colony forming cells, while silencing of EAR-2 increased colony size but not colony numbers, consistent with the notion that EAR-2 functions to limit hematopoietic differentiation. Since over-expression prevents cells from differentiating, it is expected that fewer colonies would be observed since progenitor cells simply would not differentiate into colony forming progenitor cells. On the other hand, knockdown of EAR-2 promotes differentiation in suspension culture. Hematopoietic differentiation occurs through a series of cell divisions and hence requires time in order for the differences to be visualized. Indeed, in secondary replating of shEAR-2 cells, we observe lower colony forming capability, consistent with increased differentiation to expanding progenitors and eventual loss of stem cells (Fig. 4d iv).

We do not claim, nor does the data support, that EAR-2 inhibits the differentiation of all hematopoietic lineages across the board. EAR-2 inhibits the differentiation of specific lineages at specific stages of differentiation. Here we show inhibition of differentiation of erythroid and myeloid lineages. We speculate that in the context of normal hematopoiesis, EAR-2 is a transcriptional repressor that regulates lineage bifurcation decisions by repressing genes necessary for lineage commitment and differentiation along various hematopoietic branches. It may very well be possible that while over-expression of EAR-2 inhibits differentiation of several hematopoietic lineages, other lineages, for example B220+ B cells, might be enhanced. Elucidating the precise role of EAR-2 throughout the hematopoietic hierarchy will be of great interest.

This dramatic phenotype of EAR-2 overexpression and EAR-2 shRNA may appear inconsistent with the phenotype of EAR-2 deficient animals, in which no hematopoietic phenotype is described [44, 45], but this is not the case. While it is difficult to extrapolate the in vitro findings of promotion of myeloid differentiation it would be reasonable to expect that animals with targeted deletion of EAR-2 would exhibit enhanced hematopoietic differentiation. We conjecture that EAR-2 functions as a transcriptional repressor preventing the activation of pathways necessary for lineage commitment and/or terminal differentiation. Hence, in the absence of EAR-2, repression of those pathways responsible for differentiation would not occur, therefore resulting in a phenotype where cells differentiate, as observed in the knockout animal. Without specific analysis of repopulation ability or response to stress or injury this phenotype would be difficult to distinguish from normal control animals.

Experiments using in vitro progenitor culture and adoptive transfer techniques cast light on the mechanism by which EAR-2 affects hematopoietic differentiation. We found that unregulated expression of EAR-2 in bone marrow cells impaired differentiation (Figs. 3 and 4) and extended clonogenicity (Fig. 1a and Fig. 4c). Conversely, silencing of EAR-2 expression promotes hematopoietic differentiation (Fig. 4), leading to depletion of undifferentiated (lineage negative) cells and loss of proliferative capacity (Fig. 4d iv). These effects of EAR-2 on hematopoiesis are mediated via its actions as a DNA-binding transcriptional repressor (Fig. 5). These data are consistent with a role for EAR-2 in regulation of hematopoietic cell differentiation, and demonstrate that unregulated expression of EAR-2 is pathogenic leading to impairment of terminal differentiation and dysplasia.

In the absence of ligand, nuclear receptors including EAR-2 act as transcriptional repressors by recruiting histone deacetylase (HDAC) co-repressor complexes to specific target genes [43]. Our observations that the effects of EAR-2 on myeloid differentiation are dependent on DNA binding and are abrogated by HDAC inhibition are consistent with this mechanism, suggesting a model in which EAR-2 represses the transcriptional program necessary for lineage commitment.

## Conclusions

The results reported here implicate EAR-2 as a player not only in self-renewal of normal HSCs but also as an MDS oncogene. These findings have important practical implications. Nuclear receptors are eminently “druggable” – agonist and antagonist NR ligands are of proven utility in a wide variety of human diseases. We have shown previously that silencing of EAR-2 expression by shRNA in AML cell lines causes differentiation and apoptosis [32]. Here we show that expression of EAR-2 is sufficient to initiate myeloid dysplasia that progresses to acute leukemia. These data raise the possibility that natural or synthetic antagonist ligands for EAR-2 may be found that may act as anti-leukemic therapeutics. The observation that animals with targeted deletions of EAR-2 are viable and fertile suggests that while such a drug would possess limited toxicity even though it would target both normal and MDS stem cells. Conversely, it may be possible to identify agonist EAR-2 ligands that promote HSC self-renewal in a controlled fashion – such ligands would be of enormous utility in therapeutic expansion of HSCs for autologous or allogeneic transplantation.

## Additional files

Additional file 1: **Figure S1.** EAR-2 over-expression chimeras develop myeloid dysplasia. **(a)** Percentage of transduced cells post-transplant. Mean is demarcated with a line, input percentage is shown by red triangle. **(b,c)** Photomicrographs of **c** bone marrow at low **(b)** and high **(c)** magnification demonstrates abnormal localization of immature precursors (ALIP) in EAR-2 transplants recipients. In contrast to GFP controls, where clusters of primitive cells were found only in paratrabecular locations, EAR-2 BMT recipients exhibited such clusters also in the intertrabecular region – this phenomenon, known as ALIP is seen in myelodysplastic syndrome, where it is a predictor of aggressive disease behavior. (PDF 120 kb)

Additional file 2: **Figure S4.** Over-expression of EAR-2 in vitro inhibits hematopoietic differentiation. Bone marrow transduced with either EAR-2 or empty vector (GFP) was analyzed in colony formation assay for (a) the number of cells per colony. (b) Colony formation data was consistent across mouse strain (Balb/c). Knockdown of EAR-2 in vitro increases hematopoietic differentiation. (c) Silencing of murine BM with shRNA increased colony size so dramatically that the difference was visible macroscopically and was accompanied by a noticeable change in pH. (d, e) Gene silencing depleted bone marrow in short-term culture of the lineage negative fraction, such that the cells differentiated into granulocytes. For analysis of lineage expression cells were stained with a cocktail of CD3, CD45R/B220 (RA3-6B2), CD11b (M1/70), erythroid marker (TER-119), biotin Ly-6G (RB6-8C5) and analyzed using flow cytometry on a Becton Dickinson LSR II. All samples analyzed were gated based on FSC/SSC and GFP+ cells. Panel e shows two representative fields of view. (PDF 392 kb)

## Abbreviations

5FU: 5-fluorouracil
ALIP: Abnormal localization of immature precursors
AML: Acute myeloid leukemia
BFU-E: Burst forming unit—erythroid
BM: Bone marrow
BMT: Bone marrow transplant
CFU-GM: Colony forming unit—granulocyte monocyte
CMML: Chronic myelomonocytic leukemia
EAR-2: V-erb A related-2, NR2F6
EGFP: Enhanced green fluorescent protein
FACS: Fluorescence activated cell sorting
FBS: Fetal bovine serum
G-CSF: granulocyte-colony stimulating factor
GFP: Green fluorescent protein
HDAC: Histone deacetylase
HSC: Hematopoietic stem cell
IL: Interleukin
IMDM: Iscoves Modified Dulbecco’s Medium
MDS: Myelodysplastic syndrome
PBS: Phosphate buffered saline
shRNA: Short hairpin RNA
