# Supplementary material for: The orphan nuclear receptor EAR-2 (NR2F6) inhibits hematopoietic cell differentiation and induces myeloid dysplasia in vivo
Source: Biomark Res. 2018 Dec 7;6:36. doi: 10.1186/s40364-018-0149-4 (PMC6286615; doi:10.1186/s40364-018-0149-4)
Supplement: Supplementary file 1 — Figure S1. EAR-2 over-expression chimeras develop myeloid dysplasia. (a) Percentage of transduced cells post-transplant. Mean is demarcated with a line, input percentage is shown by red triangle. (b,c) Photomicrographs of c bone marrow at low (b) and high (c) magnification demonstrates abnormal localization of immature precursors (ALIP) in EAR-2 transplants recipients. In contrast to GFP controls, where clusters of primitive cells were found only in paratrabecular locations, EAR-2 BMT recipients exhibited such clusters also in the intertrabecular region – this phenomenon, known as ALIP is seen in myelodysplastic syndrome, where it is a predictor of aggressive disease behavior. (PDF 120 kb) [file 40364_2018_149_MOESM1_ESM.pdf]

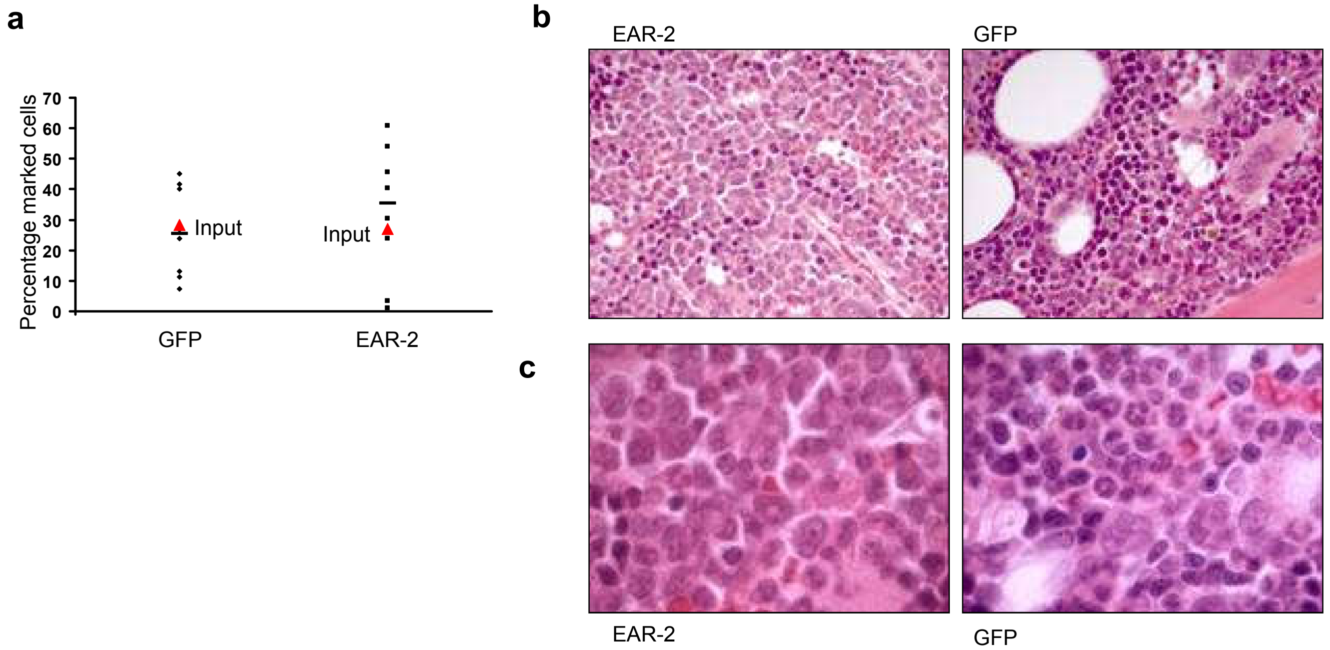

Figure S1. EAR-2 over-expression chimeras develop myeloid dysplasia. **(a)** Percentage of transduced cells post-transplant. Mean is demarcated with a line, input percentage is shown by red triangle. **(b,c)** Photomicrographs of bone marrow at low **(b)** and high **(c)** magnification demonstrates abnormal localization of immature precursors (ALIP) in EAR-2 transplants recipients. In contrast to GFP controls, where clusters of primitive cells were found only in paratrabecular locations, EAR-2 BMT recipients exhibited such clusters also in the intertrabecular region – this phenomenon, known as ALIP is seen in myelodysplastic syndrome, where it is a predictor of aggressive disease behavior
