# Supplementary material for: The orphan nuclear receptor EAR-2 (NR2F6) inhibits hematopoietic cell differentiation and induces myeloid dysplasia in vivo
Source: Biomark Res. 2018 Dec 7;6:36. doi: 10.1186/s40364-018-0149-4 (PMC6286615; doi:10.1186/s40364-018-0149-4)
Supplement: Supplementary file 2 — Figure S4. Over-expression of EAR-2 in vitro inhibits hematopoietic differentiation. Bone marrow transduced with either EAR-2 or empty vector (GFP) was analyzed in colony formation assay for (a) the number of cells per colony. (b) Colony formation data was consistent across mouse strain (Balb/c). Knockdown of EAR-2 in vitro increases hematopoietic differentiation. (c) Silencing of murine BM with shRNA increased colony size so dramatically that the difference was visible macroscopically and was accompanied by a noticeable change in pH. (d, e) Gene silencing depleted bone marrow in short-term culture of the lineage negative fraction, such that the cells differentiated into granulocytes. For analysis of lineage expression cells were stained with a cocktail of CD3, CD45R/B220 (RA3-6B2), CD11b (M1/70), erythroid marker (TER-119), biotin Ly-6G (RB6-8C5) and analyzed using flow cytometry on a Becton Dickinson LSR II. All samples analyzed were gated based on FSC/SSC and GFP+ cells. Panel e shows two representative fields of view. (PDF 392 kb) [file 40364_2018_149_MOESM2_ESM.pdf]

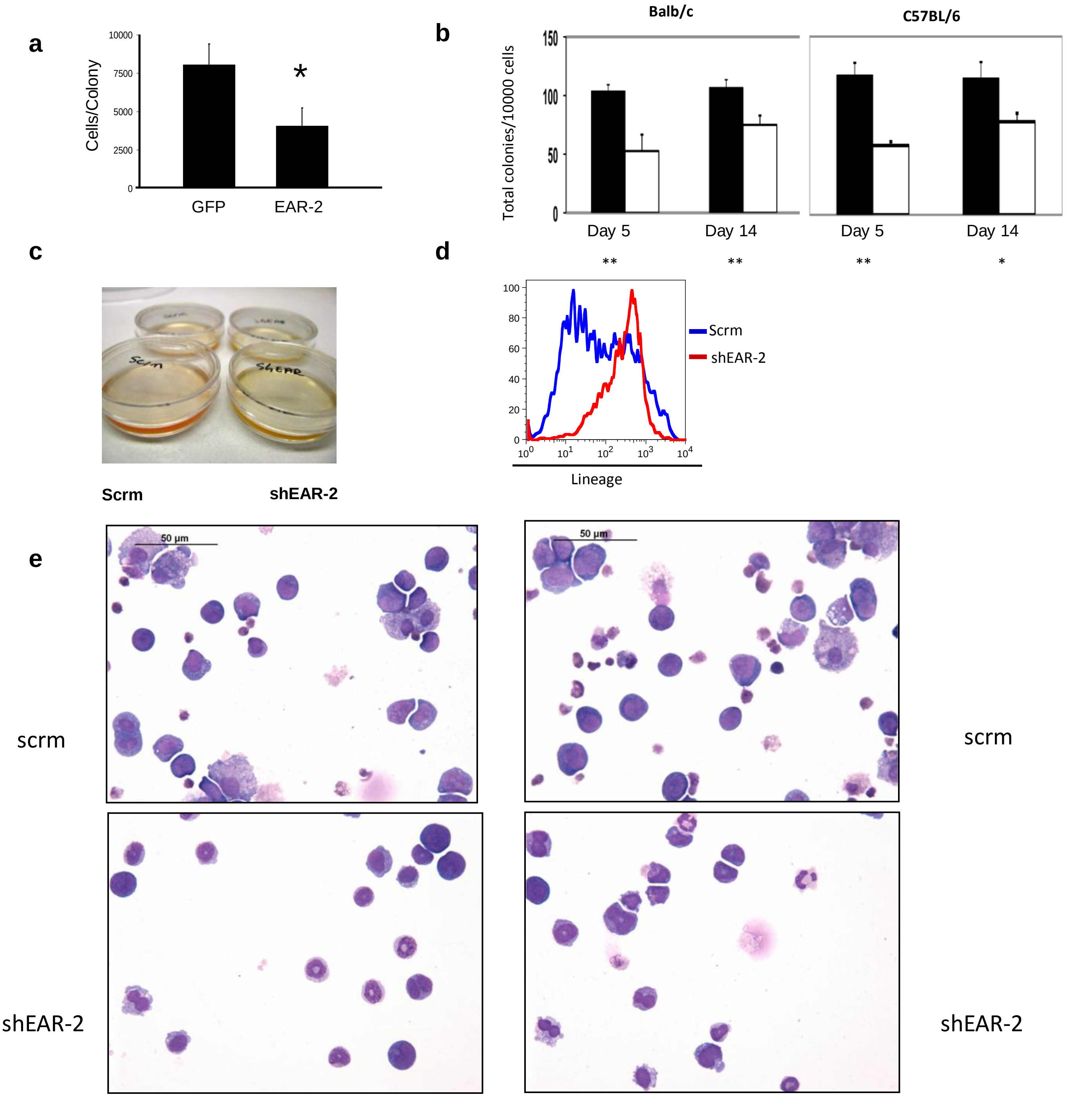

Figure S4. Over-expression of EAR-2 in vitro inhibits hematopoietic differentiation. Bone marrow transduced with either EAR-2 or empty vector (GFP) was analyzed in colony formation assay for (a) the number of cells per colony. (b) Colony formation data was consistent across mouse strain (Balb/c). Knockdown of EAR-2 in vitro increases hematopoietic differentiation. (c) Silencing of murine BM with shRNA increased colony size so dramatically that the difference was visible macroscopically and was accompanied by a noticeable change in pH. (d, e) Gene silencing depleted bone marrow in short-term culture of the lineage negative fraction, such that the cells differentiated into granulocytes. For analysis of lineage expression cells were stained with a cocktail of CD3, CD45R/B220 (RA3-6B2), CD11b (M1/70), erythroid marker (TER-119), biotin Ly-6G (RB6-8C5) and analyzed using flow cytometry on a Becton Dickinson LSR II. All samples analyzed were gated based on FSC/SSC and GFP+ cells. Panel e shows two representative fields of view.
